# The influence of life history characteristics on flea (Siphonaptera) species distribution models

**DOI:** 10.1186/s13071-016-1466-9

**Published:** 2016-03-29

**Authors:** Luther van der Mescht, Peter C. le Roux, Conrad A. Matthee, Morgan J. Raath, Sonja Matthee

**Affiliations:** Department of Conservation Ecology and Entomology, Stellenbosch University, Private Bag X1, Matieland, 7602 South Africa; Evolutionary Genomics Group, Department of Botany and Zoology, Stellenbosch University, Private Bag X1, Matieland, 7602 South Africa; Department of Plant Science, University of Pretoria, Private bag X20, Hatfield, 0028 South Africa

**Keywords:** Siphonaptera, MaxEnt, SD, Host specificity, Microhabitat preference, Life history, Small mammals, Climate envelope modelling, TSS, AUC

## Abstract

**Background:**

Ectoparasites exhibit pronounced variation in life history characteristics such as time spent on the host and host range. Since contemporary species distribution (SD) modelling does not account for differences in life history, the accuracy of predictions of current and future species’ ranges could differ significantly between life history groups.

**Results:**

SD model performance was compared between 21 flea species that differ in microhabitat preferences and level of host specificity. Distribution models generally performed well, with no significant differences in model performance based on either microhabitat preferences or host specificity. However, the relative importance of predictor variables was significantly related to host specificity, with the distribution of host-opportunistic fleas strongly limited by thermal conditions and host-specific fleas more associated with conditions that restrict their hosts’ distribution. The importance of temperature was even more pronounced when considering microhabitat preference, with the distribution of fur fleas being strongly limited by thermal conditions and nest fleas more associated with variables that affect microclimatic conditions in the host nest.

**Conclusions:**

Contemporary SD modelling, that includes climate and landscape variables, is a valuable tool to study the biogeography and future distributions of fleas and other parasites taxa. However, consideration of life history characteristics is cautioned as species may be differentially sensitive to environmental conditions.

**Electronic supplementary material:**

The online version of this article (doi:10.1186/s13071-016-1466-9) contains supplementary material, which is available to authorized users.

## Background

Ectoparasites exhibit pronounced variation in life history strategies with parasite-host associations ranging from one-to-one symbiosis (host-specific) to multi-partner symbiosis (host generalist). Furthermore, life history also differs between taxa with some parasite species being only temporarily associated with the body of a host (e.g. ticks and fleas), while others are more permanently linked with a host (e.g. lice) [1–3]. Consequently, it can be argued that the distribution of parasite species with multiple free-living stages (i.e. temporary parasites) are likely more strongly affected by the off-host environment (e.g. climatic and landscape features), whereas the distribution of permanent parasites may be indirectly driven by factors affecting host assemblages (e.g. shelter and food) (see [1, 2]). Temporary parasite taxa, however, are also characterized by varying levels of host association [1, 2] with these subtle differences in life history characteristics also potentially adding further complexity to patterns of parasite species distributions [2, 4]. These complex relationships between parasite, host and environment may make it difficult to achieve accurate range predictions for ectoparasites.

The increasing threat posed by emerging infectious diseases [5, 6] coupled with an increase in the availability of species occurrence records, has stimulated renewed interest in predicting the current and future distributions of arthropod vectors. Species distribution (SD) modelling has proved useful for this purpose, e.g. [7–9], with particular success for several medically and veterinary important arthropod vectors, e.g. [8, 10–12], including a single study on fleas [10]. In the latter study the authors used standard climatic variables (temperature, relative humidity and precipitation) in a GARP modelling approach to estimate the regional distribution of 18 flea species that act as vectors of *Yersinia pestis*, the bacterial agent of plague in California, USA. Given the recent re-emergence of certain flea-borne diseases [6] any improvements in our understanding of changes in flea vector distributions is valuable for the field of epidemiology.

Fleas are obligate ectoparasites of terrestrial vertebrates, and are regarded as "permanent satellites" of their hosts, due to the intimate association between fleas and hosts [13, 14]. In general, fleas spend part of their life-cycle in the host’s nest (egg, larvae and pupae) while adults occur on the body of the host. The length of time that adults spend on the host varies between flea taxa [1, 14, 15] and this difference in microhabitat preference allows fleas to be categorized either as “fur” (adults spend more time on the host), “nest” (adults spend more time in the nest of the host) or “fur/nest” species (adults spend more or less equal amounts on the host and in the nest of the host) [1, 14, 15]. Flea species also differ in terms of host specificity, which ranges from host-specific (recorded from ≤ 2 host species) to host-opportunistic (recorded from > 2 host species) [1, 14, 16]. There appears to be no relationship between microhabitat preference and level of host specificity exhibited by flea species (i.e. nest fleas are not generally regarded as having a higher level of host specificity). However, differences in level of host association between a flea and a host may have profound implications for the level of exposure to environmental features (e.g. climate and landscape), see [17–20], highlighting the need to assess the importance of these life history characteristics in SD modelling studies.

The aim of this study was therefore to compare SD model performance and the relative importance of predictor variables between flea species with different microhabitat preferences (fur *vs* nest) and level of host specificity (opportunistic *vs* specific). We predicted that fur fleas will be more accurately modelled due to being more strongly affected by variables associated with regional environmental conditions (e.g. climate), while nest fleas will be less accurately modelled due to being affected by conditions within the host nest (e.g. soil conditions and microclimate; since all life stages spend the majority of their life-cycle off the host and have limited dispersal capabilities). Furthermore, we predict that host-specific fleas will be more strongly associated with the abiotic variables constraining their host’s distribution and therefore will be more accurately modelled than host-opportunistic fleas. This is expected because host-specific fleas are assumed to be adapted to the immediate environment of their specific host and thus are expected to tolerate a narrower range of physical conditions compared to host-opportunistic fleas (see [14, 21, 22]).

## Methods

**Fig. 1:**
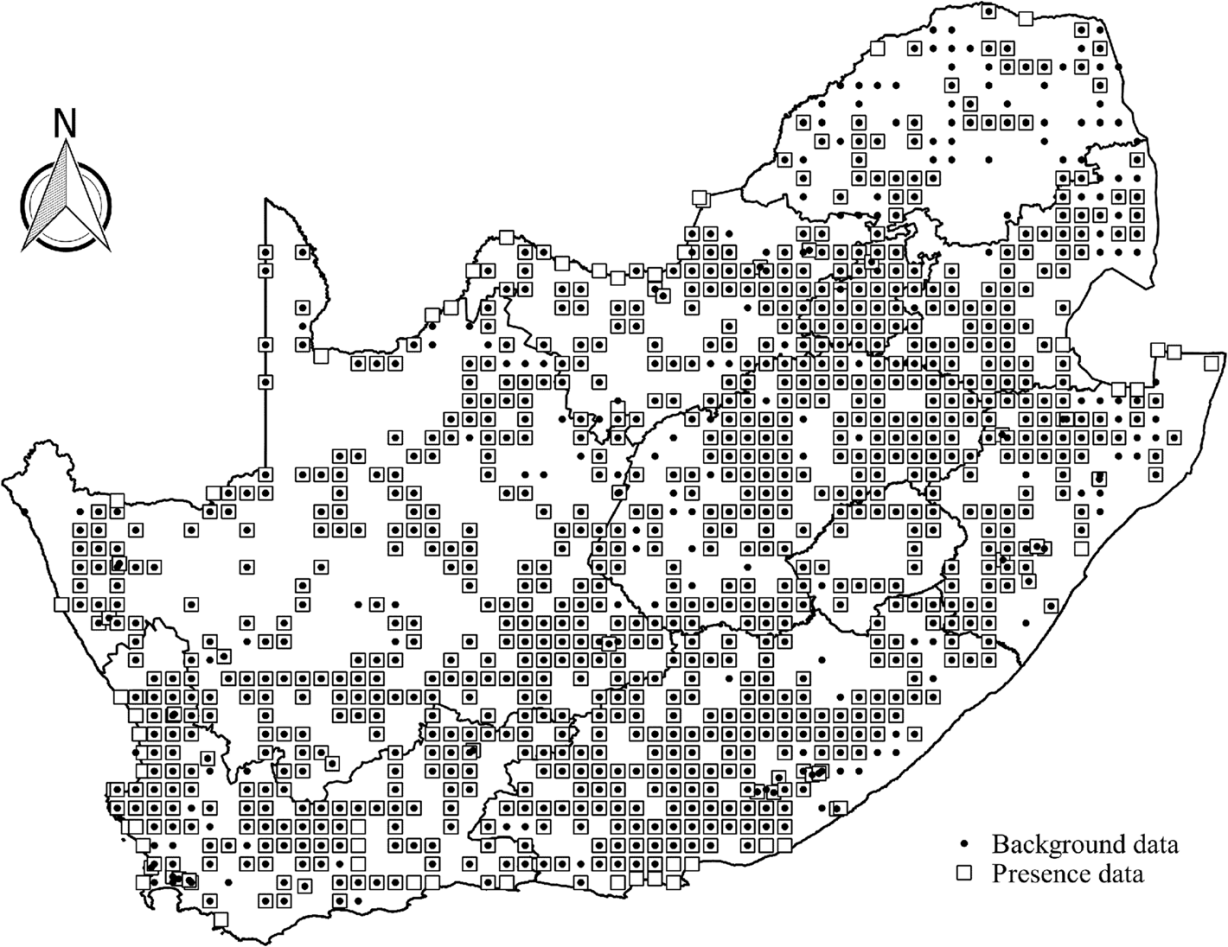
Map of South Africa indicating 1064 background and 1993 presence data points for all 21 flea species used in the flea distribution models

A dataset comprising occurrence records for flea species that parasitize 83 small mammal species (rodents, elephant shrews and shrews) from 1064 localities across South Africa were compiled from published literature [23–25] (Fig. 1). Flea species were selected for modelling when having occurrence records from ten or more localities (following [26–28] and when their microhabitat preference and host specificity are known (Fig. 1; Table 1). The raw data associated with background and presence data points are available upon request from the authors. Flea species were subsequently categorized by microhabitat preference (fur: adult stage spend more time on the host *vs* nest: adults spend more time in the nest of the host *vs* fur/nest: adults stage spend roughly equal time in the nest and on the host) [1, 14, 15] and host specificity (opportunistic: recorded from > 2 host species *vs* specific: recorded from ≤ 2 host species) ([1] based on [23, 29]) (Table 1). Fleas that spend almost equal amounts of time in the nest and fur (indicated as fur/nest in Table 1) were only included when analysing species by host specificity. Furthermore, instances where a flea species was only recorded once from a host species were seen as accidental infestations and not considered when classifying host specificity.

**Table 1:** Microhabitat preference, host specificity, number of unique occurrence records and two measures of model performance (AUC and TSS) for each flea species

Species	Microhabitat preference	Host specificity^a^	Occurrence records	AUC^b^	TSS^b^
*Chiastopsylla coraxis*	nest	opportunistic	51	0.907	0.694
*Chiastopsylla mulleri simplex*	fur/nest	specific	26	0.948	0.752
*Chiastopsylla pitchfordi*	nest	opportunistic	58	0.907	0.706
*Chiastopsylla quadrisetis*	fur/nest	specific	15	0.940	0.804
*Chiastopsylla rossi*	nest	opportunistic	88	0.545	0.067
*Ctenopthalmus calceatus*	fur/nest	opportunistic	85	0.848	0.518
*Demeillonia granti*	fur	specific	15	0.737	0.386
*Dinopsyllus ellobius*	fur/nest	opportunistic	439	0.646	0.231
*Dinopsyllus lypusus*	fur	opportunistic	34	0.894	0.545
*Epirimia aganippes*	fur	opportunistic	52	0.738	0.280
*Listropsylla agrippinae*	fur	opportunistic	150	0.760	0.360
*Listropsylla chelura chelura*	nest	specific	50	0.821	0.454
*Listropsylla dorripae*	nest	specific	70	0.695	0.277
*Listropsylla fouriei*	nest	specific	13	0.665	0.272
*Listropsylla prominens*	fur	opportunistic	28	0.882	0.519
*Praopsylla powelli*	fur/nest	specific	15	0.885	0.560
*Xenopsylla eridos*	nest	opportunistic	134	0.810	0.499
*Xenopsylla mulleri*	nest	specific	10	0.978	0.763
*Xenopsylla pirei*	nest	opportunistic	284	0.737	0.423
*Xenopsylla trifaria*	nest	specific	24	0.733	0.363
*Xenopsylla versuta*	fur/nest	specific	32	0.890	0.636

^a^Opportunistic (recorded from > 2 host species) and specific (recorded from ≤ 2 host species)

^b^AUC, area under the curve of the receiver operating characteristic (ROC); TSS, True Skill Statistic

Preliminary climate and landscape variables were selected based on our knowledge of flea ecology, limiting candidate variables to only include predictors that are considered ecologically relevant to flea species (following [30–32]). All predictor variables and flea occurrence data were converted to Quarter Degree Grid Cell (QDGC) scale and cropped to the borders of South Africa. Five remotely-sensed climate-based variables (daytime land surface temperature (hereafter referred to as day temperature), Leaf Area Index (LAI), Normalised Difference Vegetation Index (NDVI), rainfall, water vapour, and soil characteristics) and one landscape feature variable (Topography) were extracted from the NASA-NEO website (http://neo.sci.gsfc.nasa.gov/about/) as potential predictor variables (missing values were estimated as the average of contiguous cells). Climate is known to generally influence flea populations to a greater extent than host species, especially at regional and local scales [20, 33], with air temperature, rainfall and relative humidity being important for flea survival, see [1, 10, 33–36]. NDVI is widely used in arthropod vector distribution modelling and is a measure of primary productivity (plant photosynthetic activity), and therefore can be considered as a proxy for general arthropod habitat conditions [37, 38]. Furthermore, NDVI has also been successfully used in small mammal resource and population dynamics studies [38–40] and therefore may also be a surrogate for host availability. LAI is a measure of plant canopy structure and can influence incident radiation and evapotranspiration at the soil surface [41]. Additionally soil data, including soil organic carbon content, pH, cation exchange capacity, percentage sand and bulk density, were extracted from the SoilGrids database [42] (http://www.soilgrids.org/) at a depth of 60–100 cm. These soil characteristics may be expected to have direct (via microhabitat) and indirect (small mammal burrowing conditions) effects on flea species distributions [35, 43–46].

To remove collinearity between climate-based predictors and to summarize seasonality, we performed harmonic regressions for all of the climate variables using monthly data from January 2001 until June 2014 (following the methods of [47]) (see Additional file 1 for R script). These Fourier-transformed variables (“harmonic variables” hereafter) represent key temporal climate trends, reflecting different measures of seasonality [47]. Maximum, minimum, mean, range, and coefficient of variation (CV) values were also calculated for each climate variable. The correlation between all predictor variables was then calculated to identify collinear predictors, with the most strongly correlated variable excluded and the process repeated until the strongest correlation was weaker than |0.7|. When choosing between two strongly correlated variables, the preference was to drop harmonic variables rather than the other more biologically-interpretable variables (i.e. maximum, minimum, mean, range, CV, topography, and soil variables). Through this process an initial set of 68 predictor variables was reduced to 19 predictors with minimal collinearity (see Additional files 2 and 3) and with clear ecological relevance, avoiding the inclusion of variables that are irrelevant and/or will inflate models [7, 9, 47, 48]. The raw data associated with predictor variables are available upon request from the authors. All analyses were conducted in R v3.1.3 [49] and ArcGIS 10.1 [50].

Species distribution modelling relates species presence (or presence and absence) data to environmental variables to predict the distribution of species over a specified geographic range [51]. In this study MaxEnt models, based on the maximum entropy algorithm, were used with presence-only data (MaxEnt v3.3.3; [52, 53]). To account for potentially spatially-biased sampling of fleas across our study region (e.g. due to more studies being conducted in protected areas), MaxEnt models were adjusted for uneven sampling by incorporating background data reflecting patterns in sampling effort [31, 32] (see Fig. 1). All 1064 localities from which flea species occurrences have been published were therefore included as background points to distinguish false absences (lack of survey data) from true absences (species was not recorded) (Fig. 1).

For each flea species variable importance (i.e. relative contribution of each predictor variable) was calculated using the full dataset. The mean importance of each variable type (the average of all related individual predictor variables) was determined for each group of flea species (microhabitat preference and host specificity). Model performance was evaluated using 10-fold cross-validation to calculate the area under the curve (AUC) of the receiver operating characteristic (ROC; [54]) in MaxEnt and the true skill statistic calculated from MaxEnt output in R (TSS; [55], see Additional file 4). Analysis of variation (ANOVA) was used to test if AUC and TSS values differed according to species’ microhabitat preference and host specificity. Non-metric multi-dimensional scaling (NMDS) and analysis of similarity (ANOSIM; implemented from the *vegan* package in R) were used to test if differences in variable importance were related to microhabitat preference or host specificity.

## Results

A total of 21 flea species from small mammals were selected with known microhabitat preference, host specificity, and sufficient occurrence records (Table 1). Overall, model performance was highly variable between flea species (AUC from 0.545 to 0.978; TSS from 0.067 to 0.804; Table 1), but was good to excellent on average (mean ± SE, AUC = 0.799 ± 0.026; TSS = 0.464 ± 0.045).

**Fig. 2:**
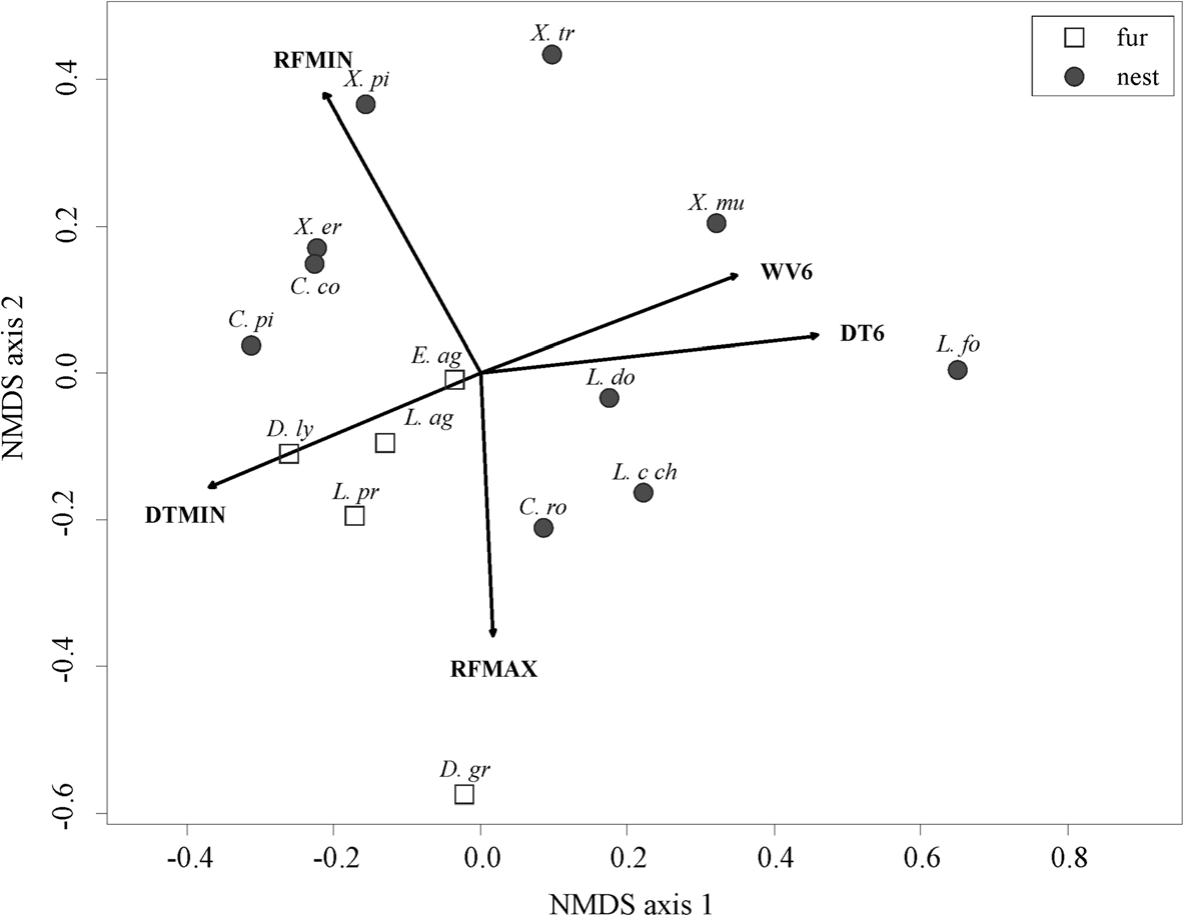
NMDS ordination plot showing the relationship between microhabitat preference of flea species and the variable importance of predictors included in each species distribution model. The best linear fit of all variables that had a significant (*P* ≤ 0.05) influence are indicated. Variable codes: DT6, 6th harmonic component of daytime land surface temperature; DTMIN, minimum daytime land surface temperature; RFMIN, minimum rainfall; RFMAX, maximum rainfall; WV6, 6th harmonic regression component of water vapour

**Fig. 3:**
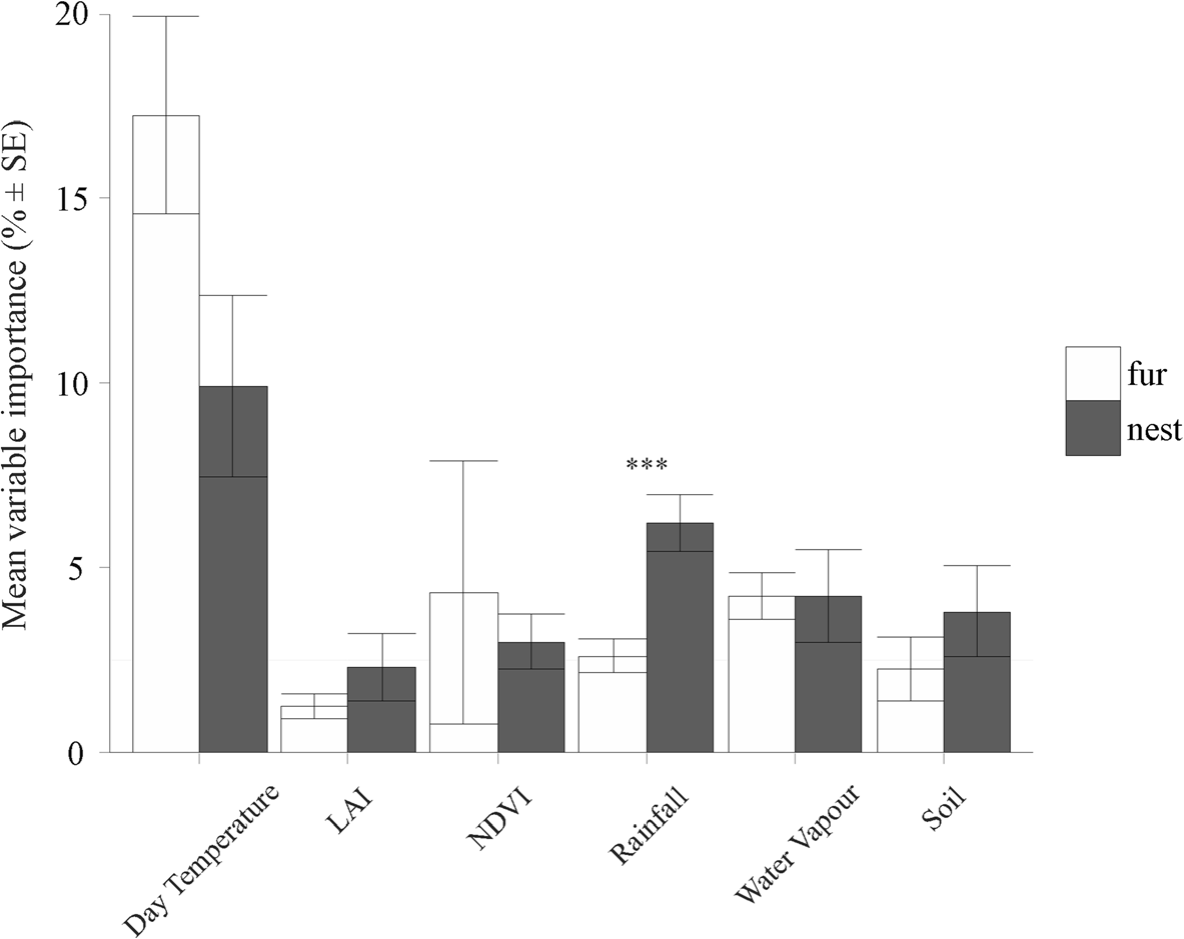
Variable importance (i.e. percent relative predictor variable type importance) in MaxEnt models, averaged across flea species for each variable type based on microhabitat preference (see Table 2 for variable category information). Significant differences in the contribution of predictor variable types between the two categories of species are indicated by asterisks: *** *P* < 0.001, ** *P* < 0.01, * *P* < 0.05

For flea microhabitat preference, there was no significant difference in AUC (*F*_*1, 13*_ = 0.120, *P* = 0.735) or TSS (*F*_*1, 13*_ = 0.101, *P* = 0.756) values between fur (AUC = 0.802 ± 0.035; TSS = 0.418 ± 0.050) and nest (AUC = 0.780 ± 0.041; TSS = 0.452 ± 0.070) fleas. In addition, importance of the predictor variables did not differ significantly between fur and nest fleas (ANOSIM: *R*^*2*^ = 8.7 %, *P* = 0.204; Fig. 2). Variable importance averaged by variable type (i.e. day temperature, LAI, NDVI, rainfall, water vapour and soil; Table 2), revealed that temperature-related variables were most important for predicting species distributions, and this was particularly so for fur fleas (Fig. 3). Rainfall was the second most important variable type for predicting the occurrence of nest fleas (having a significantly higher relative contribution to nest fleas than fur fleas; Fig. 3). Considering variables individually, minimum day temperature and minimum water vapour contributed significantly more strongly to fur than nest fleas while minimum rainfall contributed significantly more towards predicting the distribution of nest fleas than fur fleas (see Additional file 5).

**Table 2:** Final list of variable type and individual predictor variables used for modelling flea species with different life histories

Variable type	Predictor variable
Day temperature	6th harmonic component of daytime land surface temperature
	7th harmonic component of daytime land surface temperature
	Minimum daytime land surface temperature
LAI	3rd harmonic component of daily LAI
	4th harmonic component of daily LAI
NDVI	4th harmonic component of daily NDVI
	7th harmonic component of daily NDVI
Rainfall	4th harmonic component of daily rainfall
	6th harmonic component of daily rainfall
	7th harmonic component of daily rainfall
	Minimum rainfall
	Maximum rainfall
Water vapour	4th harmonic component of daily water vapour
	5th harmonic component of daily water vapour
	6th harmonic component of daily water vapour
	Minimum water vapour
Soil	Soil percentage sand
	Soil organic carbon
	Soil pH

**Fig. 4:**
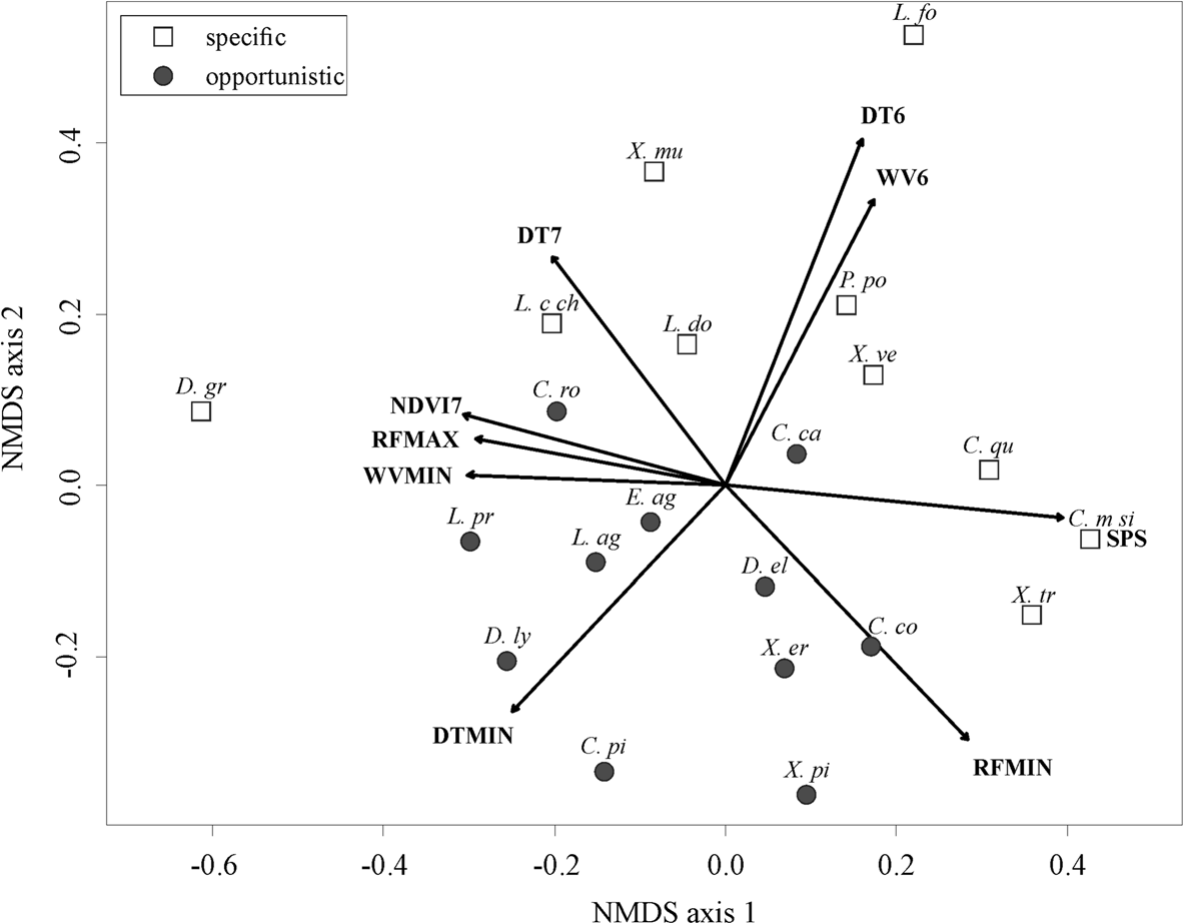
NMDS ordination plot showing the relationship between host specificity of flea species and the variable importance of predictors included in each species distribution model. The best linear fit of all variables that had a significant (*P* ≤ 0.05) influence are indicated. Variable codes: DT6, 6th harmonic component of daytime land surface temperature; DT7, 7th harmonic component of daytime land surface temperature; DTMIN, minimum daytime land surface temperature; NDVI7, 7th harmonic component of NDVI; RFMIN, minimum rainfall; RFMAX, maximum rainfall; WV6, 6th harmonic regression component of water vapour; WVMIN, minimum water vapour; SPS, soil percentage sand

**Fig. 5:**
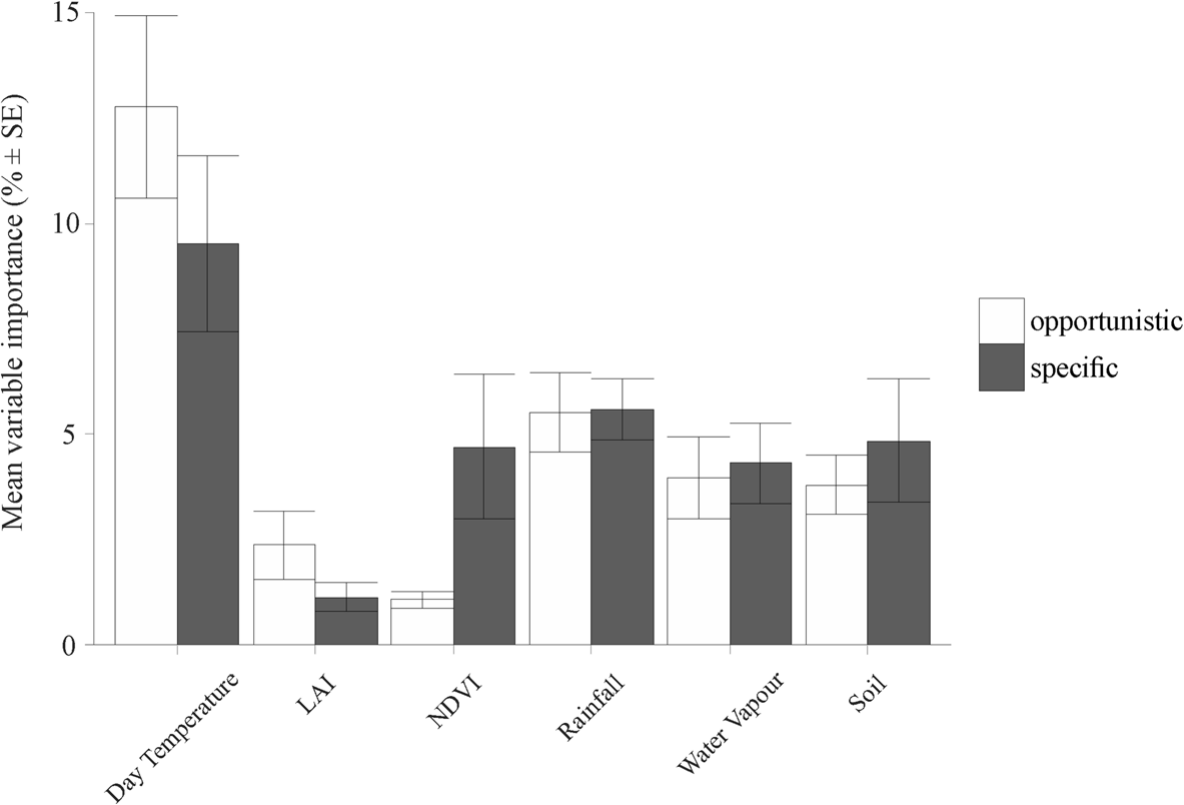
Variable importance (i.e. percent relative predictor variable type importance) in MaxEnt models, averaged across flea species for each variable type based on host specificity (see Table 2 for variable category information). Significant differences in the contribution of predictor variable types between the two categories of species are indicated by asterisks: *** *P* < 0.001, ** *P* < 0.01, * *P* < 0.05

In the case of host specificity, there was no significant difference in AUC (*F*_*1, 24*_ = 0.427, *P* = 0.520) or TSS (*F*_*1, 24*_ = 0.699, *P* = 0.411) values between host-opportunistic (AUC = 0.789 ± 0.035; TSS = 0.440 ± 0.059) and host-specific (AUC = 0.829 ± 0.036; TSS = 0.527 ± 0.065) species. However, there was a strong and significant difference in variable importance between host-opportunistic and host-specific fleas (ANOSIM: *R*^*2*^ = 31.3 %, *P* = 0.003; Fig. 4). When averaging variable importance by type, day temperature, followed by rainfall, was the most important predictor for both host-opportunistic and host-specific fleas. However, day temperature contributed more strongly towards host-opportunistic than host-specific fleas, whereas rainfall contributed equally towards both host-opportunistic and host-specific fleas (Fig. 5). Further, NDVI contributed more strongly towards host-specific than host-opportunistic fleas (Fig. 5). Considering variables individually, minimum day temperature contributed significantly more towards host-opportunistic than host-specific fleas (see Additional file 5).

## Discussion

The study confirms that contemporary SD modelling, that includes climatic and landscape variables, has potential for improving predictions of changes in the distribution of flea species. The model performance for fleas was good overall, with life history not having a significant effect on model performance. However, the importance of predictor variables differed considerably between species with different life history strategies, suggesting differential sensitivity to climate (temperature and rainfall) and landscape feature (NDVI) variables among groups of fleas.

Abiotic conditions such as air temperature, relative humidity and precipitation are important for fleas [1, 10, 33–36] especially at the regional scale [20, 56]. In particular, air temperature and relative humidity can have a direct (influence on egg production and rate of oviposition, development and survival of pre-imaginal fleas, and survival of imagoes) [34–36] and/or indirect affect (influence hosts abundance and distribution) on flea survival [1, 14, 20]. However, flea species also vary in terms of preferred temperature range (reviewed by [14]) and although there is evidence that phylogenetic relatedness may explain this pattern for certain flea taxa it appears not to be consistent across all taxa [14, 20, 57]. In the present study we found that temperature is an important predictor of flea species ranges, irrespective of life history, but that the relative importance of temperature differed between flea life histories. In particular, the higher importance of temperature for host-opportunistic compared to host-specific fleas may be related to their physiological limits [34–36]. Host-opportunistic fleas generally have a wider tolerance range (climate and host composition), are geographically more widespread and are likely to experience more environmental heterogeneity throughout their geographical range [14, 20–22]. In contrast, host-specific fleas generally have narrower tolerance ranges (climate and host composition), are less widespread and are likely to experience more environmental homogeneity throughout their geographical range [14, 20–22]. Thus, although temperature is important, especially for the development of immature nest stages in general, it is evidently less important for host-specific fleas in this study.

Host-specific fleas seems to be more influenced by variables related to host availability as evident from the higher NDVI importance for host-specific compared to host-opportunistic fleas in the current study. NDVI has been used as a measure of the amount of suitable habitat for arthropod vectors [37, 38] and may also be a good proxy for several aspects of habitat quality that are of relevance to fleas. Abiotic factors influence vegetation and thus food supply [58–62] and actual or perceived predation risk [63–65] which will affect small mammal host abundance and distribution [38–40], this in turn may directly influence flea abundance and distribution [20]. In the current study, environmental stability could facilitate small mammal host population stability and subsequent flea population stability and specialization [66, 67], because host-specific fleas tolerate restricted abiotic and biotic conditions [68]. In contrast, host-opportunistic fleas can tolerate variable environments (e.g. seasonal environments that have greater variability in NDVI) that could facilitate less stable small mammal host populations and are thus less affected by NDVI [38].

Predictor importance for individual variables also differed between species with different microhabitat preferences. Temperature was again overall the most important predictor for both fur and nest fleas. However, the difference in the importance of temperature was even more pronounced when compared with level of host-specificity (as discussed above), with temperature being more important for fur fleas as opposed to nest fleas. Fur fleas spend more time on the body of the host and as a result are potentially exposed to higher fluctuations in ambient temperature and relative humidity compared to nest fleas (see [46]). It has been suggested that nest fleas may have evolved to spend more time in nests due to more constant and buffered microclimatic conditions brought about by the physical properties of nests [1, 69, 70]. Although discrepancies in the fur versus nest dichotomy are mainly attributed to ambient temperature in the literature (see [14] and references within), it has also been suggested that within-host among-flea difference can be explained by this dichotomy, whereas between-host within-flea differences are better explained by between-host difference in nest construction [71, 72].

Studies on different nest types (burrows, above-ground nests and nest within rock crevices) all demonstrate that microclimatic conditions are more stable in nests compared to the external environment [43–46]. Other factors that can facilitate higher and more stable humidity levels in the nest include, the presence of nest material [46, 73] and higher soil water capacity of the mineral and organic enriched nest soil [74], due to the activities of hosts in nests [46, 73]. In support of this, rainfall contributed significantly more towards explaining the distribution of nest compared to fur fleas in the current study. Rainwater naturally filters down into soil layers which can contribute to maintaining higher and more stable humidity levels in the nest of hosts [43, 45, 46]. The type and complexity of host nests are influenced by the soil texture [46, 75]. It is therefore not surprising that soil was more important for nest compared to fur fleas. It is evident from this study that the interplay between temperature, rainfall and potentially relative humidity with nest construction can facilitate the separation of fleas into different microhabitat types.

Our study suggests that SD modelling can be a useful tool for studying the drivers of flea species distributions and also the underlying ecology of these species, but caution needs to be taken when deciding which predictor variables to include. While our results highlight how contemporary models can perform well, it is unclear to what extent the inclusion of biotic interactions (e.g. host availability and competition) could further improve model accuracy and transferability [7, 56]. Specifically, modelling and comparing flea species with different levels of host-specificity could possibly benefit from including accurate host species occurrence data. Furthermore, it is important to remember that ecological patterns are affected by processes that act at different scales [76]. For example, the assembly of flea compound (all species infesting a host community) communities is strongly affected by host filters (e.g. evolutionary, biogeographic and historical forces) at the continental scale, while at the regional and local scale it is more strongly affected by the abiotic filters (e.g. topography, NDVI, and climate) (see [17–20]. Therefore, the value of the inclusion of host species data (and the predominant importance of temperature in our models) may be contingent on the spatial scale of analysis.

## Conclusions

Five of the flea species in our study (*Chiastopsylla rossi*, *Dinopsyllus lypusus*, *Listropsylla dorripae*, *Xenopsylla pirei*, and *Xenopsylla versuta*) have been implicated as possible vectors of diseases in South Africa (e.g. plague [29]). As a consequence, accurate forecasts of the future distributions of these species are valuable for the field of epidemiology. Our results suggest that despite differences in their degree of host specificity, SD models should perform well for all of these species. However, due to differential sensitivity to different groups of climatic and landscape variables, host-specific and generalist flea species are likely to respond very differently to changes in abiotic conditions. As a result, our results suggest the importance of explicitly considering species life history as a potential mediating variable when predicting flea species distributions.

## Additional files

Additional file 1: Calculation of coefficients of harmonic regression for climate variables and topography in R. Description of data: R script illustrating the production of the coefficients of harmonic regression for climate variables and topography. (R 3 kb) Additional file 2: Collinearity among the 19 final individual predictor variables. All final predictor variables had correlation values below 0.7 or above−0.7. Description of data: The table contains collinearity among final 19 predictor variables chosen for modelling. (XLSX 14 kb) Additional file 3: List of predictor variables indicating their respective reference codes. Description of data: The table contains a list of all predictor variables considered for this study and their abbreviations as reference codes. (XLSX 11 kb) Additional file 4: Calculation of the true skill statistic (TSS) for each replicate of the 10-fold cross-validation from MaxEnt output in R. Description of data: R script illustrating the calculation of the true skill statistic (TSS) for each replicate of the 10-fold cross-validation from MaxEnt output. (R 2 kb) Additional file 5: Variable importance (i.e. percent relative predictor variable individual contribution) in MaxEnt models, averaged across flea species based on (a) microhabitat preference and (b) host specificity (see Additional file 2 for variable reference code). Significant differences in the contribution of predictor variables between the two categories of species are indicated by asterisks: *** *P* < 0.001, ** *P* < 0.01, * *P* < 0.05. Description of data: The bar plot figure illustrate variable importance (i.e. percent relative predictor variable individual contribution) in MaxEnt models, averaged across flea species based on microhabitat preference and host specificity. (BMP 7730 kb)
