# Supplementary figures and images for: The influence of life history characteristics on flea (Siphonaptera) species distribution models
Source: Parasit Vectors. 2016 Mar 29;9:178. doi: 10.1186/s13071-016-1466-9 (PMC4812659; doi:10.1186/s13071-016-1466-9)

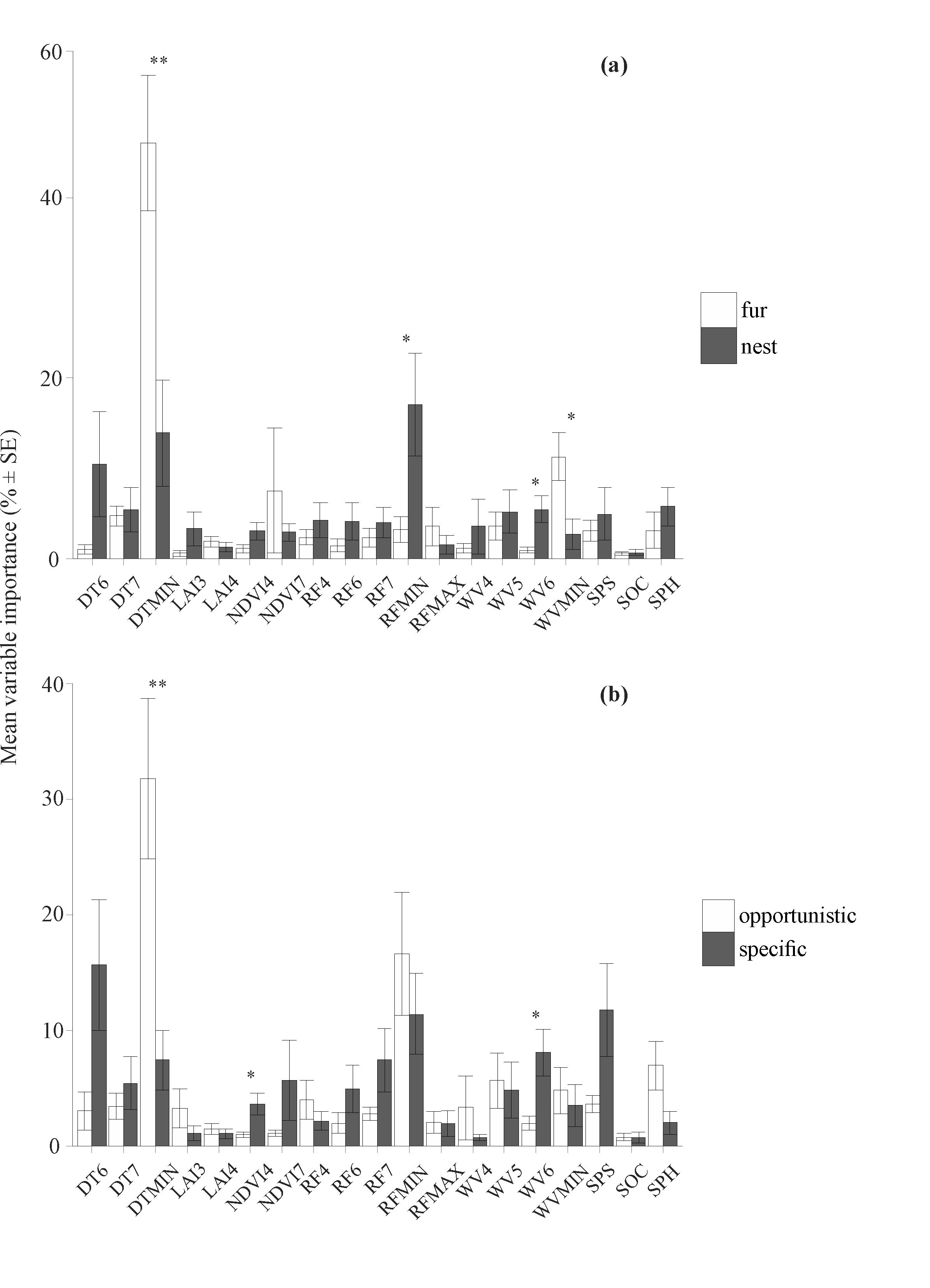

Supplement: Additional file 5: — Variable importance (i.e. percent relative predictor variable individual contribution) in MaxEnt models, averaged across flea species based on (a) microhabitat preference and (b) host specificity (see Additional file 2 for variable reference code). Significant differences in the contribution of predictor variables between the two categories of species are indicated by asterisks: *** P < 0.001, ** P < 0.01, * P < 0.05. Description of data: The bar plot figure illustrate variable importance (i.e. percent relative predictor variable individual contribution) in MaxEnt models, averaged across flea species based on microhabitat preference and host specificity. (BMP 7730 kb) [file 13071_2016_1466_MOESM5_ESM.bmp]
